# A Simple and Rapid LC-MS/MS Method for the Determination of BMCL26 a Novel Anti-Parasitic Agent in Rat Plasma

**DOI:** 10.4172/2155-9872.1000266

**Published:** 2015-08-15

**Authors:** Ramakrishna R Voggu, Xiang Zhou, Bin Su, Baochuan Guo

**Affiliations:** Department of Chemistry, Cleveland State University, USA

**Keywords:** BMCL26, Anti-parasitic, LC-MS/MS, Protein precipitation, Rat plasma

## Abstract

BMCL26 is a potential drug derived from nimesulide, which has exhibited the substantial anti-parasitic activity in various cell lines. To conduct various pharmacological and toxicological properties of this drug, we developed and validated a rapid LC-MS/MS method for its quantification in accordance with the FDA guidelines. Protein precipitation with 0.1% formic acid in acetonitrile was used to extract the analytes along with the internal standard (JCC76) from rat plasma. It was found that the calibration curve of the method had an excellent linearity (r^2^ ≥ 0.9993) for the analyte concentration ranging from 0.5 to 100 ng/mL with acceptable inter- and intra-assay, precision, accuracy and stability. The matrix effect and extraction recovery were in the range of 101.30–110.10% and 90.16– 105.00%, respectively. This LC-MS/MS method is simple and rapid and can be used in the future pharmaceutical studies of BMCL26.

## Introduction

Human African trypanosomiasis, also known as sleeping sickness, is a vector-borne parasitic disease and a serious health threat to a large number of people living in sub-Saharan Africa, where health systems are challenged at best [1–3]. *Trypanosoma brucei gambiense* (*T. b. gambiense*) and *Trypanosoma brucei rhodesiense* (*T. b. rhodesiense*) are the etiological parasites that cause sleeping sickness in humans. These parasites live and grow extracellularly in the blood and tissue fluids of humans or cattle and are transmitted among hosts by tsetse flies (*Glossina* spp.). Without effective treatment, the disease can lead to coma and ultimately death. If the patients do not receive treatment in a timely manner, the neurological damage caused by these parasites is irreversible even after treatment [3,4]. Current drugs used to treat human trypanosomiasis include Suramin, Pentamidine, Melarsoprol and Eflornithine [5], however, these drugs do not effectively treat the disease, maintaining an urgent need for new, more effective and less expensive drugs for the treatment of human African trypanosomiasis [4–6].

Tubulin is a very attractive target in the field of anti-cancer drug discovery, and several successful tubulin binders are used clinically as first-line chemotherapeutic agents [7]. Tubulin also plays an essential role during trypanosome cell division. The fast, population-doubling rate of trypanosomes makes them highly dependent on tubulin polymerization/depolymerization [8]. More importantly, tubulin is critical for trypanosome locomotion, which is essential for trypanosomes survival. Tubulin inhibitors not only block *T. brucei* cell division but also affect the locomotive functions of flagellum and lead to cell death [9]. Some microtubule-disrupting herbicides, such as phosphoric thioamide herbicide Amiprophos-Methyl (APM) and dinitroaniline herbicides, exhibit activity against protozoan parasites by targeting tubulin [10–14]. Research works have optimized these compounds, generating more potent and selective tubulin inhibitors for *T. brucei* [10]. Webovertz’s group successfully developed several drug candidates that show promising anti-parasite activity and selectivity *in vitro*. However, these compounds did not show substantial potency *in vivo* due to their poor stability [15].

Recently, we have developed a class of tubulin inhibitors as anti-cancer agents [16,17]. These compounds share the same core scaffold and bind to a colchicine-binding domain on tubulin [16]. We performed *T. brucei* cell-growth inhibition assays with our compounds, some of which exhibited very specific inhibitory effects on *T. brucei* growth resulting in selectivity indices (IC_50_ inhibiting human cancer cell growth/IC_50_ inhibiting *T. brucei* cell growth) of 5 or more. Among these compounds, BMCL26 has been identified as a potential drug candidate. BMCL26 exhibited activity against *T. brucei* cell proliferation with an IC50 of 1.62 µM but inhibited mammalian cell growth with an IC_50_ of 55.35 µM [18]. The selective index of BMCL26 is approximately 34, and its IC_50_ (for inhibiting *T. brucei* proliferation) is in the low micromolar range, which is obtainable in blood. The present investigation addresses the critical need of developing a method to accurately quantify BMCL26 concentrations in blood. Thus, in this paper, we report the development and validation of a robust and highly sensitive LC-MS/MS method for quantitating BMCL26 in rat plasma with an LLOQ of 0.5 ng/mL and a linear calibration range of up to 100 ng/mL.

## Experimental

### Chemicals and reagents

**Figure 1:**
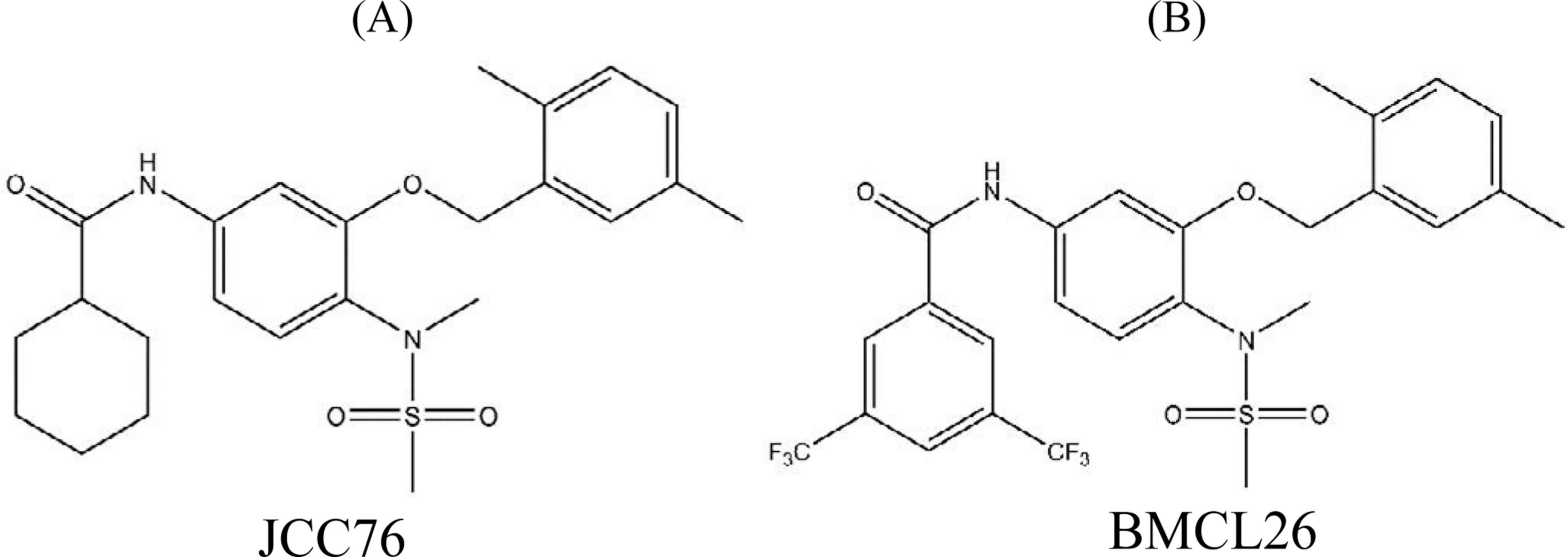
The chemical structures of JCC76 (A), internal standard BMCL26 (B).

BMCL26 and JCC76 (internal standard) were synthesized and purified according to previously published procedures [18,19]. Methanol (HPLC grade) and acetonitrile were from Pharmco-Apper (Philadelphia, Pennsylvania, USA). Formic acid and ammonium acetate (analytical grade) were purchased from Sigma Aldrich Chemical Company (Allentown, Pennsylvania, USA). Deionized water was obtained using a Barnstead Nano pure water purification system with a Nanopure Diamond Pack Organic free DI cartridge from Thermo Scientific (Waltham, Massachusetts, USA). Six individual lots of rat plasma (Sprague-Dawley rat plasmas K2) were obtained from Innovative Research (Novi, Michigan, USA) (Figure 1).

### Calibration standards and quality-control samples

#### Preparation of stock and working solutions

A set of BMCL26 working solutions containing 10, 20, 50, 150, 400, 1000 and 2000 ng/ mL were prepared by serial dilution using methanol and 1 mg/mL stock solution. The 150 ng/mL working solution of JCC76 (IS) was diluted from a stock solution of 1 mg/mL in methanol. Stock solutions and working solutions were stored at −20°C and 4°C.

### Calibration and preparation of quality-control (QC) plasma samples

Calibration plasma samples were prepared by spiking 10 µl of corresponding BMCL26 working solutions in 200 µl of rat plasma (mixture of 6 lots) with drug concentrations of 0.5, 1.0, 2.5, 5, 12.5, 25, 50, and 100 ng/mL. QC samples at three concentrations, 1.25 (low), 10 (mid) and 80 (high) ng/mL, were prepared by adding 10 µL of the appropriate BMCL26 working solution and 200 µL of drug-free plasma. Calibration and QC samples were frozen at −20°C overnight and then treated using the following sample preparation procedure before LCMS/ MS analysis.

### Sample extraction

QC samples and blanks were removed from the −20°C freezer and thawed to room temperature. Ten µl of JCC76 working solution were spiked into each 200 µL aliquot of plasma calibrators/QCs/blanks, excepting the double blank, into which 10 µL of acetonitrile was added. The solutions were then vortexed immediately for 30 sec, after which each sample was deproteinized by adding 800 µl of 0.1% formic acid in acetonitrile, sonicating for 15 minutes, and centrifuging at 13,000 × g for 15 minutes. The supernatants were then transferred into auto sampler vials for LC-MS/MS analysis.

### LC-MS/MS analysis

LC-MS/MS analysis was performed using a 5500 Q-TRAP triple quadrupole, tandem mass spectrometer (AB Sciex, Toronto, Canada) coupled with an Electrospray Ionizer (ESI) operated in negative ion mode (Framingham, Massachusetts, USA) and interfaced with High Performance Liquid Chromatography (HPLC, Shimadzu, Columbia, Maryland, USA) system that uses auto-sampling and online vacuum degassing. All data acquisition and processing were conducted using Analyst software, version 1.5.2 (AB Sciex).

**Figure 2:**
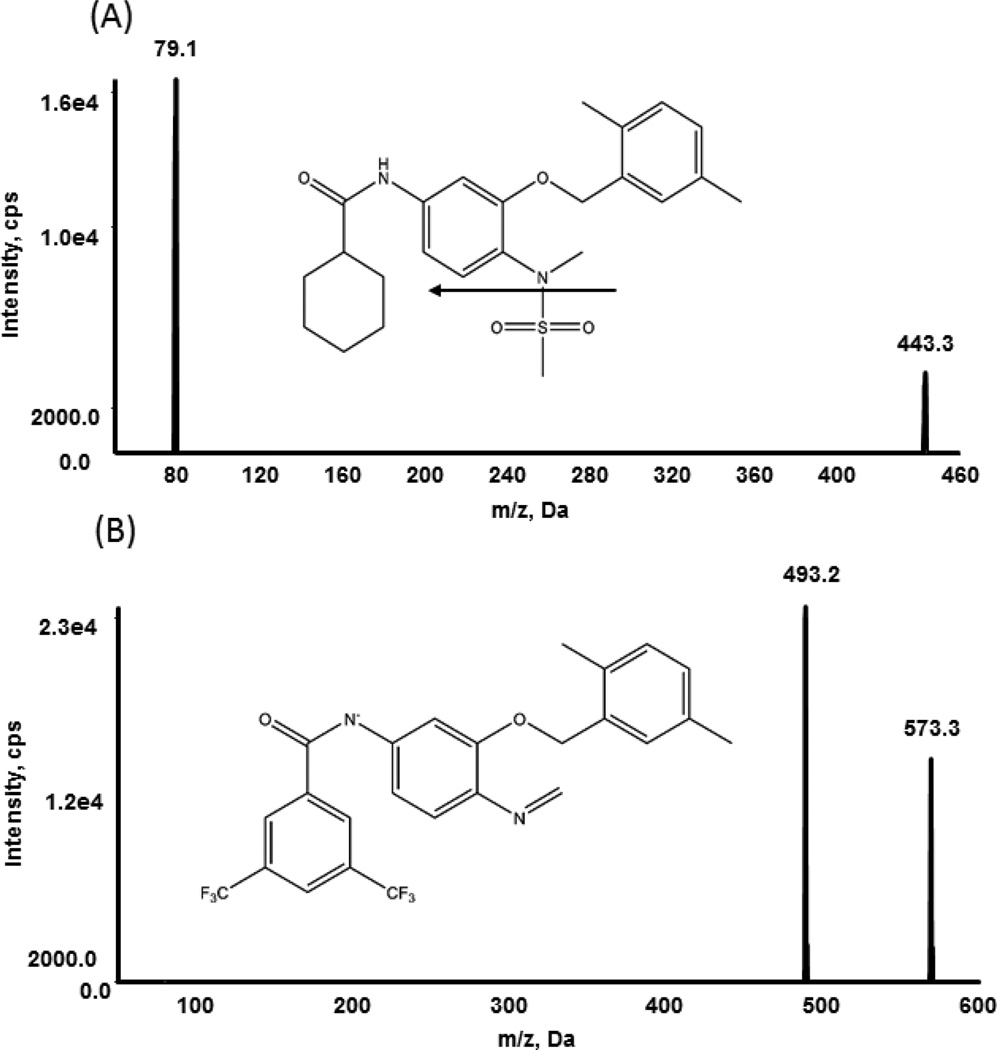
Precursor/product ion spectra and proposed fragmentation pathways for internal standard JCC76 (A) and analyte BMCL26 (B).

Analytical separation of BMCL26 was achieved using a Luna C8 (2) HPLC column (50 × 2.0 mm, 5 micron) with a C8 security-guard cartridge from Phenomenex (Torrance, California, USA). Mobile phase A contained 50 µM ammonium acetate in 2% Methanol, and mobile phase B contained 50 µM ammonium acetate in 90% Methanol. Sample aliquots of 5 µl were injected onto the column and eluted via the following gradient flow: 0–0.6 min, 70% B, 1.6 min, 90% B, 7.5 min, and stop (Table 1). The column was equilibrated for 0.5 min before each run. Negative electrospray ionization (ESI) mode was selected, and the MRM (multiple reaction monitoring) function was used for quantification, with the transitions set at m/z 573.3 → 493.2 for BMCL26 and m/z 443.2 → 79.1 for JCC76 (IS) (Figure 2), respectively. The following ion-source-dependent parameters were used: nebulization gas (30), heating gas (30), curtain gas (35), ion spray voltage (−4330 ev) and temperature (500°C). Compounddependent parameters were manually optimized as follows: declustering potential, −40; entrance potential, −10; collision energy and cell exit potential for both analyte and internal standard, −30, −100, −13, and −9.

### Analytical method validation

The LC-MS/MS assay was fully validated according to the Food and Drug Administration (FDA) Bioanalytical Method Guidelines [20] and other references [21–23]. The entire assay was validated for linearity, accuracy, precision, selectivity, extraction recovery, matrix effect and stability.

### Linearity and calibration

Eight concentrations of BMCL26 (0.5, 1.0, 2.5, 5, 12.5, 25, 50, and 100 ng/mL) were selected for the plasma calibration curve (n=2 for each of the eight calibrators, average for each calibrator plotted). A weighed linear regression, using 1/x as the weighing factor, was used to calculate the slope and correlation coefficient of the calibration curve.

### Accuracy and precision

To determine the intra- and inter-day accuracy and precision of the assay, we tested five replicates of BMCL26 at the LQC, MQC, and HQC using 1.25, 10 and 80 ng/mL. Intra-assay and inter-assay precisions were defined as Relative Standard Deviations (RSD) between replicate measurements. Accuracies were calculated using the following formula: accuracy (%)=(experimental concentration - spiked concentration)/ (spiked concentration) × 100. The criteria for data acceptability included accuracy and precisions within ± 15% of the nominal value and the RSD.

### Extraction recovery and matrix effects

The extraction recoveries and matrix effects of BMCL26 for three concentrations of QC samples (low, 1.25 ng/mL; medium, 10 ng/ mL; and high, 80 ng/mL) were determined by comparing peak areas of analyte-spiked plasma aliquots before extraction to peak areas of analyte-spiked solutions extracted from blank plasma and comparing the response of solutions spiked with analyte after extraction to the response of analyte dissolved in the mobile phase.

### Stability studies

The stabilities of BMCL26 in rat plasma samples were determined using two distinct QC standards (1.25 and 80 ng/mL), which were tested after 8 hours at room temperature and after two months at −20°C; freeze-thaw stabilities were determined using three freeze-thaw cycles over a three day period. Short-term stabilities were also determined for post-preparation QC standards stored at room temperature for 10 hours. The QC results obtained after storage were compared with spiked concentration values by determining the percentage ratios of experimental values divided by spiked values.

The stabilities of stock and working solutions for both analyte and internal standard were also evaluated. Stock solutions of analyte and IS were stored at −20°C for 9 months. From both stored and fresh stock solutions, two QC standards (1.25 and 80 ng/mL) were prepared for the analyte, to which 7.5 ng/mL IS (JCC76) was added, and the experimentally determined concentrations of BMCL26 and JCC76 were compared (n=3 for each).

## Results and Discussion

### Optimization of mass spectrometric conditions for quantitation

The negative ionization mode was selected for both BMCL26 and JCC76 (IS) detection in this study because the analyte produced a much stronger signal for negative ionization compared to positive ionization. It was found that analyte and IS solutions prepared in methanol-water (9:1, v/v) yielded stronger signals compared to solutions prepared in acetonitrile-water (9:1, v/v). Adding ammonium acetate to the methanol-water solution substantially increased BMCL26 signals. Therefore, a gradient flow of methanol-water-ammonium acetate was chosen for the HPLC mobile phase. Figure 2 showed the parent ion spectra for both BMCL26 and IS. The highest fragment-ion signals were obtained by fine-tuning the collision energy, spray voltage, and ion source temperature. Based on our ionization and fragmentation optimization results, we chose MRM transitions of m/z 573.2 → 493.2 for BMCL26 and 443.2 → 79.1 for IS for quantification, as these product ions yielded strong and stable signals.

### Optimization of HPLC conditions

**Figure 3:**
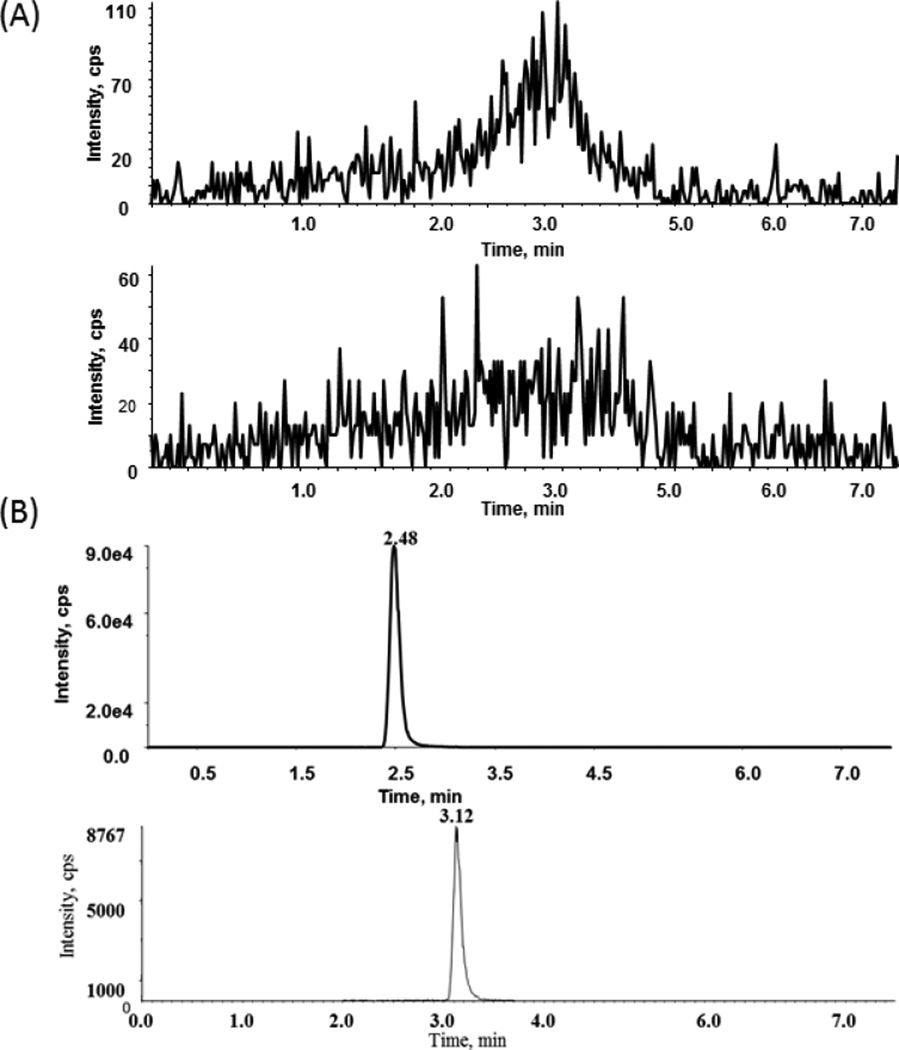
(A) MRM chromatograms of blank rat plasma in both IS and Analyte windows (B) IS JCC76 (10 ng/ml, 3.12 min) and BMCL26 at LLOQ level (0.5 ng/ml, 2.48 min).

The gradient flow of the mobile phase, which was used with different flow rates, was as follows: A: 50 µM ammonium acetate in 2% methanol and mobile phase; B: 50 µM ammonium acetate in 90% methanol (Table 1). Because the C-18 column had a carry-over problem with BMCL26, a C-8 column was used instead. The C-8 column effectively resolved the carryover problem and also yielded a better peak shape. Thus, BMCL26 and IS were separated using a C-8 column with a flow rate of 0.25 mL/ min for 3.5 min, after which the flow rate was increased to 0.6 mL/ min for column clean up and re-equilibration. Retention times were observed at 2.51 min for BMCL26 and 3.14 min for IS (Figure 3). The total run time was 8 min.

### Linearity, sensitivity, selectivity and LLOQ

Using the concentrations of BMCL26 in the working solutions, plasma calibration curves were constructed over a concentration range of 0.5–100 ng/mL. Linearity results showed a quadratic fit for BMCL26 using an eight-point calibration curve (0.5, 1, 2.5, 5, 12.5, 25, 50, and 100 ng/mL) with JCC76 (7.5 ng/mL) as the internal standard in the plasma samples. Excellent linearity was obtained with a correlation coefficient (r^2^) of 0.9993. The linear regression equation was y=0.073×−0.0085. This method exhibited high selectivity and displayed no interfering peaks in six different blank plasma samples from different sources. Using the calibration curve, the LLOQ of the method was determined to be 0.5 ng/mL. The accuracy and precision were determined for each lot of plasma at the LLOQ. The data were summarized in Table 2.

### Accuracy and precision

Intra- and inter-assay accuracies (%RE) and precisions (%CV) were evaluated by analyzing five replicates of low, medium, and high QC standards. As summarized in Table 3, the assay’s intra- and interday relative errors were 0.62 and 11.36%, respectively, and the assay’s intra- and inter-day precisions were 0.84–3.47%, respectively. These values were within acceptable limits according to FDA guidelines.

### Extraction recovery and matrix effects

It was found that protein precipitation was a good way to extract BMCL26 from plasma. Initially, we used 80% acetonitrile to precipitate plasma proteins, but a strong matrix effect was observed. Consequently, we utilized pure acetonitrile to precipitate proteins with 0.1% formic acid (for deproteinization). The latter precipitation conditions eliminated the matrix effect and achieved symmetrical chromatographic peak shapes. The absolute and relative extraction recoveries were in the range of 90.16–105.00%. The results were summarized in Table 4. As shown in Table 5, the absolute and relative matrix effects of BMCL26 from six different rat plasma samples at three QC concentrations (low (1.25 ng/mL), medium (10 ng/mL), and high (80 ng/mL) ranged from 101.30–110.10%.

### Stability

The stability of BMCL26 was determined by comparing the mean peak area ratios of BMCL26 to IS in low (1.25 ng/mL) and high (80 ng/ mL) QC samples to those of freshly prepared QC solutions (containing the same concentrations), expressed in terms of recovery. As shown in Table 6, the receives of LQC and HQC samples were 103.46–103.20%, 98.13–105.20%, and 101.86–107.54% for bench top conditions, after 3 freeze-thaw cycles and post extraction at room temperature for 10 hours, respectively. The stabilities of working solutions of BMCL26 and internal standard (JCC76), stored at 4°C for at least 6 months, were determined to be 99.60–105.00% and 115.60% for the two QC standards tested (1.25 and 80 ng/mL), to which 7.5 ng/mL IS was added (using the stored stock solution). These stability results showed no significant deviations in BMCL26 quantification under the experimental conditions used.

## Conclusion

This innovative method offers several advantages for assaying BMCL26 in rat plasma including simplicity, cost effectiveness, accuracy and precision were below 11% and 3%, with high sensitivity and selectivity. To our knowledge, this is the first time this method was used. This assay employed a simple protein precipitation procedure for plasma sample preparation. The LLOQ was as low as 0.5 ng/mL. The results from the validation study illustrated that this method can be used to determine the pharmacological and toxicological profiles of BMCL26 in rats in the future.

## Figures and Tables

**Table 1:** HPLC gradient program.

Minutes	Event	Parameters
0–0.6	B%	70 (isocratic)
0.6–1.6	B%	70–90 (liner)
0–3.5	Total flow	0.25 ml/min
3.5–3.7	Total flow	0.25–0.6 ml/min
1.6–5.5	B%	90 (isocratic)
5.5–5.6	B%	90–70 (liner)
3.5–5.9	Total flow	0.6 ml/min
5.9–6.0	Total flow	0.25–0.6 ml/min
5.6–7.5	B%	70 (liner)

**Table 2:** Accuracy and precision of BMCL26 calibration standards in rat plasma (n=5, pooled plasma samples).

Nominal Concentration (ng/mL)	Determined Concentration (ng/mL)	Accuracy (%RE)	Precision (%CV)
0.5	0.47 ± 0.03	−7.46	5.59
1	0.99 ± 0.02	−0.22	2.09
2.5	2.54 ± 0.05	1.40	2.01
5	5.14 ± 0.27	2.80	5.24
12.5	12.75 ± 0.31	2.00	2.41
25	25.27 ± 0.73	1.08	2.90
50	51.03 ± 0.97	2.06	1.90
100	98.26 ± 0.99	−1.74	1.01

**Table 3:** Inter and intra-assay accuracy and precision of BMCL26 in rat plasma.

Intra-assay						Inter-assay				
Normal (ng/ml)	Determined (ng/ml)	Accuracy (%RE)	SD	Precision (%CV)		Determined (ng/ml)	Accuracy (%RE)	SD	Precision (%CV)
1.25	1.20	11.36	0.02	1.72	1.36		9.12	0.04	3.38
10	10.20	2.00	0.16	1.64	10.20		2.00	0.35	3.43
80	80.70	0.87	0.68	0.84	80.50		0.62	2.80	3.47

**Table 4:** Absolute and relative Extraction recovery of BMCL26 in rat plasma.

Concentration of QC samples(ng/ml)	Absolute		Relative	
1.25	**Mean extraction recovery**	**%CV**	**Mean extraction recovery**	**%CV**
	104.00 ± 4.89	4.70	98.10 ± 9.73	9.92
10	105.00 ± 4.99	4.75	95.96 ± 3.61	3.76
80	96.30 ± 3.00	3.12	90.16 ± 2.87	3.18

**Table 5:** Absolute and relative matrix effect of BMCL26 in rat plasma.

Concentration of QC samples(ng/ml)	Absolute		Relative	
1.25	**Mean matrix effect**	**%CV**	**Mean matrix effect**	**%CV**
	105.00 ± 1.07	1.02	108.00 ± 5.15	4.77
10	107.00 ± 6.06	5.66	110.10 ± 8.99	8.17
80	105.00 ± 3.86	3.68	101.30 ± 4.78	4.72

**Table 6:** Stability of BMCL26 in plasma samples.

Storage conditions	Concentration (ng/ml)	Recovery %
Bench top (8 hr)	1.25	103.46
At room temp	80.00	103.20
Freeze thaw (3 cycles)	1.25	98.13
	80.00	105.20
Post extraction	1.25	101.86
(10 hr) at room temp	80.00	107.54

## Abbreviations

%CV: Coefficient of Variation
IS: Internal Standard
LLOQ: Lower Limit Of Quantification
MRM: Multiple Reaction Monitoring
AMP: Aminoprophos-Methyl
JCC76: N-(3-(2, 5-dimethylbenzyloxy)-4-(methylmethylsulfonamido)phenyl)cyclohexanecaboxamide
%RE: Relative Error
RME: Relative Matrix Effect
QC: Quality Control
SD: Standard Deviation
